# Oleanolic Acid-amino Acids Derivatives: Design, Synthesis, and Hepatoprotective Evaluation In Vitro and In Vivo

**DOI:** 10.3390/molecules23020322

**Published:** 2018-02-02

**Authors:** Fuhao Chu, Wenxi Zhang, Wenbo Guo, Zhaoyi Wang, Yuqin Yang, Xinyu Zhang, Kang Fang, Mengmeng Yan, Penglong Wang, Haimin Lei

**Affiliations:** School of Chinese Pharmacy, Beijing University of Chinese Medicine, Beijing 102488, China; chufhao@163.com (F.C.); zhangwxnn@126.com (W.Z.); wb_guo@126.com (W.G.); zywang6834@126.com (Z.W.); yangyq5@163.com (Y.Y.); xinyums@126.com (X.Z.); 18361466994@163.com (K.F.); yanmengmeng@bucm.edu.cn (M.Y.)

**Keywords:** OA-amino acids derivatives, hepatoprotective, hepatic stellate cells, apoptosisa, acidic extracellular microenvironment

## Abstract

Activated hepatic stellate cells (HSCs) are the main extracellular matrix (ECM)-producing cells in the injured liver and the key mediators of liver fibrosis; they also promote the progression of hepatocellular carcinoma (HCC). In the acidic extracellular microenvironment of HCC, HSCs are activated to promote the migration of HCC cells. It is worth attempting to alter the weak acidic microenvironment to promote activated HSC apoptosis to treat liver fibrosis and liver cancer. In the present study, a series of novel OA-amino acids analogues were designed and synthesized to introduce different amino acids in the 3-hydroxyl of OA using the ester condensation reaction to enhance hydrophilicity, alkalinity, and biological activity. We found that OA-lysine derivative (**3g**) could improve the hydrophilic of OA and induce HSCs apoptosis via inducing MMP depolarization and increasing intracellular Ca^2+^ levels. Additionally, **3g** displayed a better hepatoprotective effect than OA (20 mg/kg, intragastric administration) against the acute liver injury induced by carbon tetrachloride (CCl_4_) in mice. The results suggested that basic amino acids (lysine) could effectively enhance OA’s hydrophilicity, alkalinity, and hepatoprotective activity in vitro and in vivo, which might be likely associated with increasing bioavailability and altering an extracellular weak acidic microenvironment with further verification. Therefore, the OA-lysine derivative (**3g**) has the potential to be developed as an agent with hepatoprotective activity.

## 1. Introduction

Hepatitis, liver fibrosis or cirrhosis, and liver cancer are the interconnected liver diseases, seriously affecting the quality of human life. After acute liver injury (e.g., viral hepatitis and chemical-induced), the parenchymal cells could regenerate to substituting necrotic or apoptotic cells, which are related to the inflammatory response and limited extracellular matrix (ECM) deposition [1]. With the long-term occurrence of liver injury and inflammation, the deposition of ECM is excessively accumulated, leading to liver fibrosis or cirrhosis, the end stage of liver fibrosis, which in turn increases the risk of hepatocellular carcinoma (HCC) [2,3]. Activated hepatic stellate cells (HSCs) are the main ECM-producing cells in the injured liver and the key mediators of liver fibrosis [4]. In general, Extracellular pH (pHe) of solid tumor is acidic owing to excessive glycolysis to maintain its adequate energy supply [5]. It is found that in the tumor tissues of HCC patients, HSCs were activated under the extracellular acidic microenvironment. In turn, activated HSCs could promote the progression of HCC by creating the immunosuppressive microenvironment, promoting angiogenesis or inducing proliferation [6]. Thence, the reduction of activated HSCs is a treatment that is worth considering for liver fibrosis and HCC, and the potential agents targeting HSCs have been explored [7]. 

Oleanolic acid (3β-hydroxyolean-12-en-28-oic acid, OA) is a ubiquitous pentacyclic triterpenoid, widely found in several dietary and medicinal plants, and it is also selected as an index component in quality evaluation of Chinese herbal medicines (CHM). It is well known that OA has significant hepatoprotective activity for acute chemically induced liver injury, chronic liver fibrosis, and cirrhosis [8,9,10,11]. As an active ingredient form CHM, OA is also applied for the preparation of proprietary Chinese medicines for liver protection, such as the Ganxile Capsule, the Gansukang Capsule, the Shanzhatiaozhong Pill, and the Dangfeiliganning Capsule, etc. [12]. Due to its favorable properties, OA is considered as a base molecule for further synthetic modifications to develop lead compounds for hepatoprotective agents in our reported studies and others [13,14,15,16,17,18,19], and some of these derivatives have been therapeutic candidates in clinical trials [20]. However, there are some shortcomings in poor water-solubility, low bioavailability, and weak bioactivity, which seriously restrict their further clinical application. Amino acids are the most basic material composed of proteins in organisms and related to life activities. When amino acids or peptides were introduced into the structure of the active ingredients from traditional Chinese medicine, it could improve the hydrophilicity, increase the bioactivity, and mitigate the adverse reaction significantly [21,22,23,24,25,26,27]. In our previous study, different polarity amino acids or dipeptides were introduced into 3-hydroxyl o Oleanolic acid-ligustrazine derivative (TOA) and Betulinic acid-ligustrazine derivative (TBA), the anticancer lead compound [28,29,30]. It was found that small molecule amino acids or dipeptides could improve their hydrophilic and bioactivity [18,23,25]. 

In the present study, a series of novel OA-amino acids analogues were designed and synthesized to introduce different amino acids in the 3-hydroxyl of OA using the ester condensation reaction. Their ClogP values were calculated with the free online simulation software. The biological activity was evaluated on three pathologic live cells (HSCs, HepG2, Bel-7402) and a normal live cell (L02), respectively. Cell morphology changes on HSCs induced by **3g** were observed by Giemsa staining and DAPI staining. The apoptosis, mitochondrial membrane depolarization (MMP), and intracellular calcium levels on HSCs induced by **3g** were detected by flow cytometry. Liver morphological changes and the ability of AST and ALT in serum were examined to estimate their hepatoprotective activity on CCl_4_ acute liver injury mice model.

## 2. Results

### 2.1. Chemical Synthesis

**Scheme 1:**
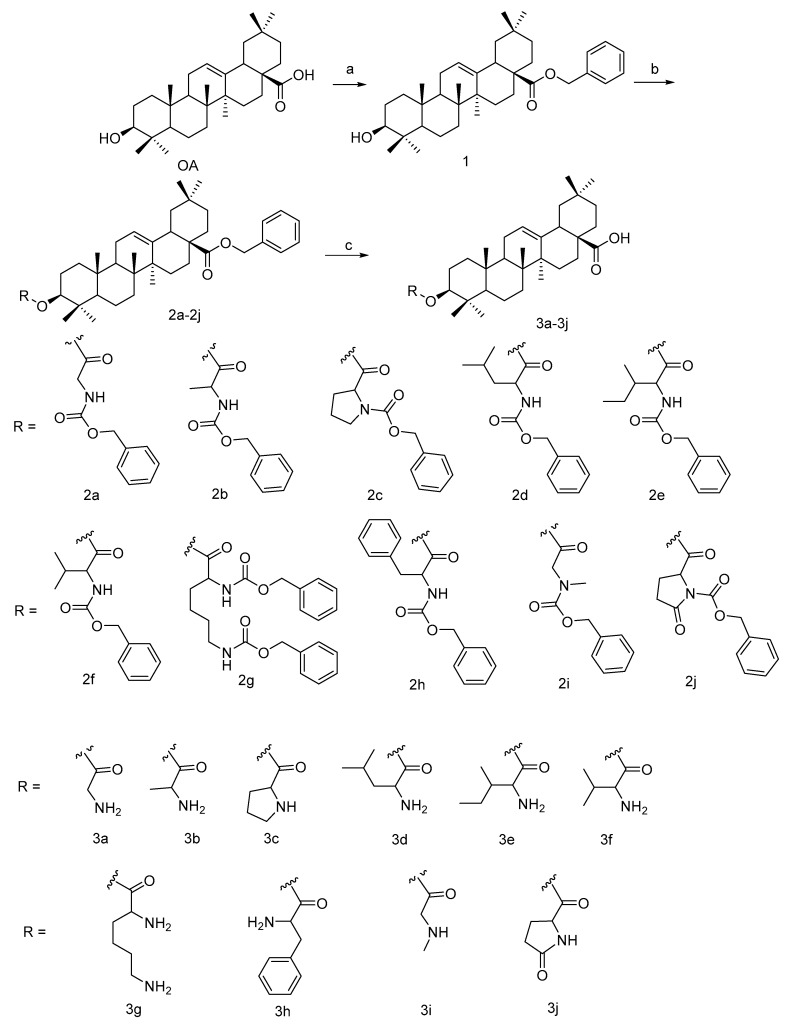
Synthesis of compounds **3a**–**3j**. Reagent: (**a**) benzyl bromide, K_2_CO_3_, DMF, reflux 2 h, 85 °C; (**b**) Cbz-(L-)-amide acids, EDCI, DMAP, dry DCM, r.t. 12 h; (**c**) Pd(OH)_2_/C, H_2_, MeOH, r.t. 12 h.

The synthetic route of target compounds **3a**–**3j** was illustrated in Scheme 1. OA was treated with benzyl bromide, potassium carbonate solution, in DMF at 85 °C for 2 h to obtain a good yield of compound **1**. The commercially available Cbz-(L-)-amide acids were coupled with compound **1** catalyzed by EDCI/DMAP in dry DCM for ester condensation to get compounds **2a**–**2j**. Then, Cbz- and benzyl- group were de-protected with Pd(OH)_2_/C simultaneously, and target compounds **3a**–**3j** were gained. The structures of all target derivatives were confirmed by ^1^H-NMR, ^13^C-NMR, and HR-MS analysis.

### 2.2. Simulation Online of ClogP

**Figure 1:**
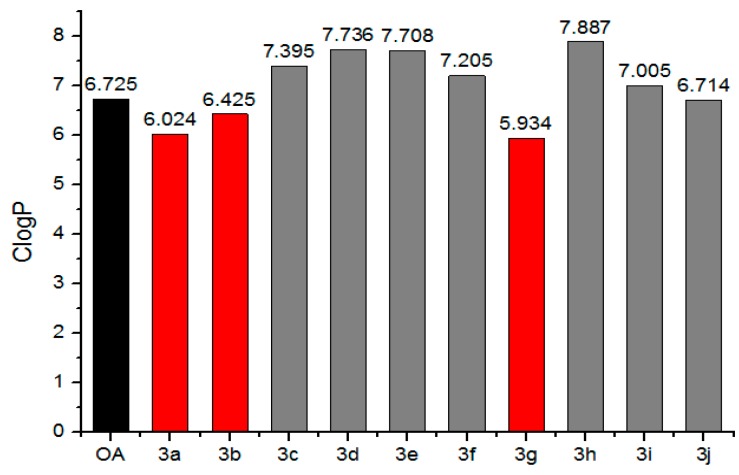
The ClogP values online simulation results of OA-amino acid derivatives.

Lipophilicity, defined by the octanol-water partition coefficient (logP), is used in drug design as a key factor related to the cell transmembrane transport [31]. In this work, the calculated logP (ClogP, the measure of hydrophobicity) values of OA-amino acids derivatives were calculated using the free online simulation software [32]. As shown in Figure 1, **3a**, **3b** and **3g** possessed lower ClogP values compared with OA. It was suggested that glycine, alanine, and lysine could improve OA’s hydrophilicity to a certain extent, which was consistent with previous report [25].

### 2.3. In Vitro Cytotoxicity

Their biological activities were evaluated on three pathologic live cells (HSCs, HepG2, and Bel-7402) and a normal live cell (L02), respectively, by MTT assays. As shown in Table 1, the IC_50_ values revealed that most of the conjugates exhibited more inhibitory activities against HSCs, HepG2, Bel-7402 than L02. Among them, compound **3g** was the most potent (IC_50_ for HSCs is 3.46 ± 1.16 µM). The structure-activity relationship analysis found that the activity was a certain negative correlation with the side chain complexity of α-carbon, while it was a positive correlation with the number of aminos. It indicated that after introducing amino acids into the 3-hydroxyl of OA, it relatively enhanced the basicity of the derivatives and improved their cytotoxicity at the same time. It further verified that small molecule amino acids (glycine and alanine) and basic amino acids (lysine) could enhance OA’s bioactivity, which was in line with our previous report [23,25].

### 2.4. Morphological Changes

**Figure 2:**
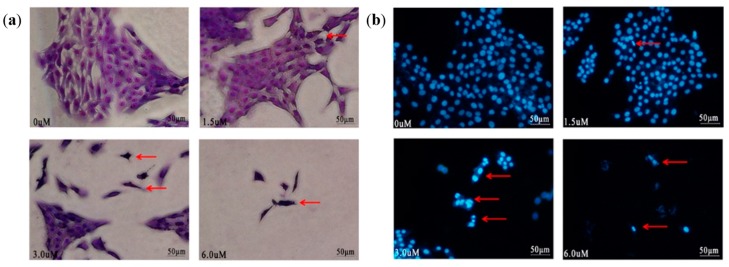
(**a**) The apoptotic morphological changes of HSCs induced by **3g** (0, 1.5, 3.0, 6.0 µM) observed by Giemsa staining; (**b**) effects of **3g** (0, 1.5, 3.0, 6.0 µM) on HSCs nuclei observed by DAPI staining.

The apoptotic morphological changes of HSCs induced by **3g** were determined under inverted phase-contrast microscope using Giemsa staining. It showed that the cells in control group appeared to have normal cellular morphology, whereas **3g** treatment caused evident nuclear fragmentation, chromatin condensation, and margination in a dose-dependent manner (as the red arrow marks in Figure 2a), all of which were characteristic morphological alterations associated with apoptosis.

Nuclear fragmentation was visualized by DAPI staining of apoptotic nuclei. It further inferred that HSCs treated with **3g** displayed hallmarks of apoptosis, such as nuclear condensation and fragmentation, as well as the formation of apoptotic bodies with irregular shape, while in the control group they appeared as normal (as shown in Figure 2b).

### 2.5. Apoptosis Analysis Using Annexin V-FITC/PI Staining

**Figure 3:**
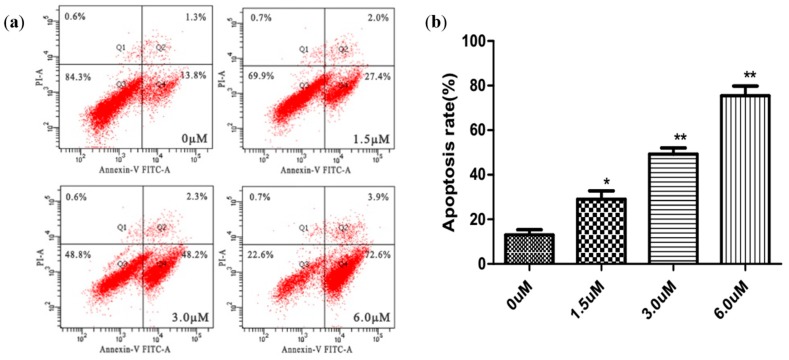
(**a**) Analysis of HSCs apoptosis using annexinV-FITC/PI staining. (**b**) apoptosis rate of **3g** (0, 1.5, 3.0, 6.0 µM) on HSCs. Values are expressed as the mean ± SD in each group (*n* = 3). * *p* < 0.01, ** *p* <0.001 vs. 0 µM.

The annexin V-FITC/PI binding assay was performed to substantiate the ability of **3g** to induce HSCs apoptosis. As shown in Figure 3, when treated with different concentrations of **3g**, the percentages (Q2 + Q4) of apoptotic HSCs increased from 15.1% in control cells to 29.4%, 50.5% and 76.5%, respectively. Consequently, the result demonstrated that **3g** could induce HSCs significant apoptosis in a concentration-dependent manner.

### 2.6. Measurement of Mitochondrial Membrane Potential (MMP)

**Figure 4:**
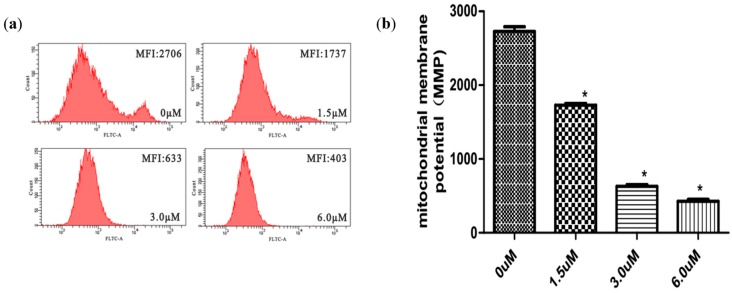
(**a**) Changes in mitochondrial membrane potential of HSCs induced by **3g**. (**b**) mitochondrial membrane potential values of **3g** (0, 1.5, 3.0, 6.0 µM) on HSCs. Values are expressed as the mean ± SD in each group (*n* = 3). * *p* < 0.001 vs. 0 µM.

To estimate the role of mitochondria in **3g**-induced HSCs apoptosis, we measured mitochondrial membrane potential changes quantitatively by the fluorescent dye Rh123. After HSCs treated with different concentrations of **3g**, the mean fluorescent intensity (MFI) decreased with the administration of higher concentrations (Figure 4), which could be related to depolarization of the membrane. The disruption of MMP indicated that **3g** could induce HSCs apoptosis via a mitochondrial apoptotic death signal pathway.

### 2.7. Measurement of Intracellular Ca^2+^ Concentrations

**Figure 5:**
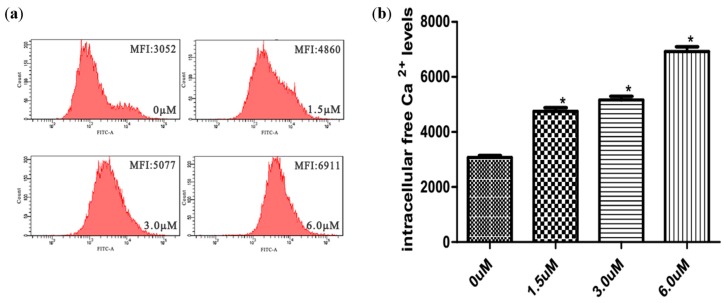
(**a**) Effect of 3g on intracellular-free Ca^2+^ levels in HSCs. (**b**) intracellular-free Ca^2+^ levels of **3g** (0, 1.5, 3.0, 6.0 µM) on HSCs. Values are expressed as the mean ± SD in each group (*n* = 3). * *p* < 0.001 vs. 0 µM.

Fluo-3AM was used to examine the effects of **3g** on intracellular Ca^2+^ mobilizations in HSCs. As the concentration of **3g** increased, intracellular-free Ca^2+^ fluorescence increased dramatically (Figure 5). It was in accordance with the tendency of MMP and cell apoptosis. It indicated that the increase of intracellular-free Ca^2+^ destructed the MMP, thus resulting in the release of mitochondrial membrane apoptin and cell apoptosis.

### 2.8. In Vivo Activity on CCl_4_ Acute Liver Injury Mice Model

Anti-hepatic fibrosis activities of OA and its derivative (**3g**) were evaluated on CCl_4_ acute liver injury mice model. Liver morphological changes and the ability of AST and ALT in serum were determined to assess significant anti-hepatic fibrosis activity of the derivatives.

#### 2.8.1. Morphological Observation by HE Staining

**Figure 6:**
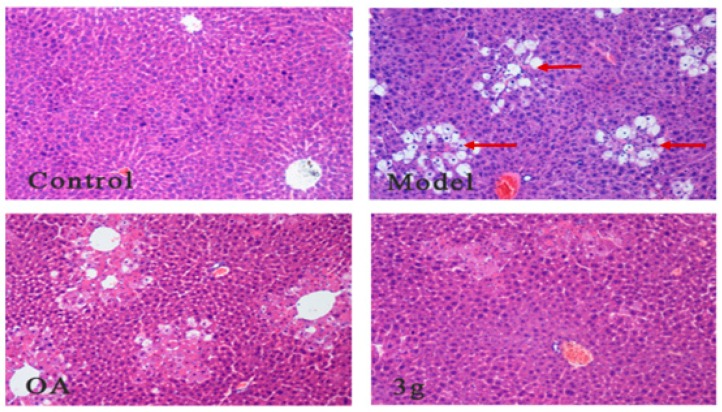
Morphological observation under an optical microscope (×100) by HE staining.

Observation under an optical microscope by HE staining; it found that liver cells are arranged neatly with uniform size, with the nucleus located in the central nucleus of the hepatocytes with a clear and rounded border in the control group. In the model group, the hepatocytes were arranged irregularly and loosely connected among the cells with obvious edema and balloon-like changes (as the red arrow marks in Figure 6), and large numbers of inflammatory cell infiltrations occurred in the portal area. After administration of **3g**, more pathological changes were relieved in balloon-like hepatocyte edema and interstitial inflammatory cell infiltration than in OA. It indicated that **3g**, OA-Lysine conjugate, had better protective activity than OA on CCl_4_ acute liver injury.

#### 2.8.2. Determination of the Ability of ALT and AST in Serum

**Figure 7:**
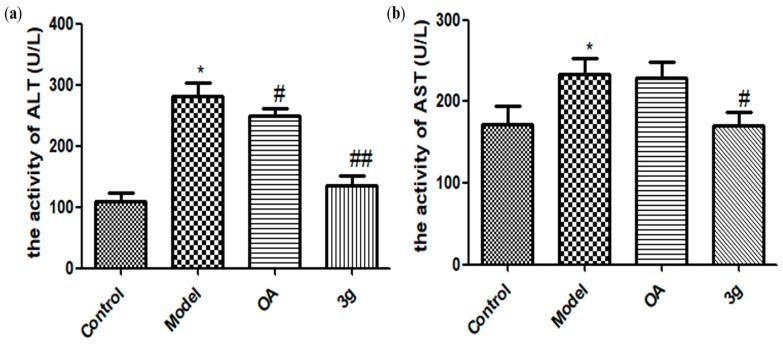
The activity of ALT (**a**) and AST (**b**) on CCl_4_ acute liver injury mice model after administration of **OA** and **3g**. Values are expressed as the mean ± SD in each group (*n* = 12). * *p* < 0.01 vs. Control, ^#^ *p* < 0.01, ^##^ *p* < 0.001 vs. model.

To assess significant hepatoprotective effect of the derivatives **3g**, the ability of ALT and AST in serum was determined with assay kit. As shown in Figure 7, compared to the control group, the activity of ALT and AST in serum increased significantly in the model group. After administration of OA and **3g**, the activity of ALT and AST reduced to some extent; in particular, the effect of **3g** was remarkably closer to control. Thence, the results suggested that **3g** had a significant effect of reducing liver damage and improving liver function.

## 3. Discussion

Many factors might induce liver injury, such as hepatitis B and C viral infection, toxic chemicals, alcoholic abuse, hepatic fat accumulation, and so on [33]. Thereafter, hepatic stellate cells (HSCs) are activated to generate excessive extracellular matrix (ECM) deposition at liver damage location, resulting in liver fibrosis without effective treatment and evolving into cirrhosis that increases the risk of liver cancer [34]. Although there has been no standard treatment for liver fibrosis until now, grabbing activated HSCs as target cells to reduce the secretion of excessive ECM could be a treatment that is worth considering for liver fibrosis. HSCs are the predominant progenitor of stromal cells in liver. It was reported that the number of activated HSCs was positively associated with the acidification level in liver cancer patient’s tumor tissues [6]. With the rapid growth and proliferation, the pathologic live cells metabolize exuberantly and consume large amounts of energy, thereby generating certain acidic metabolites (CO_2_ and lactic acid) under aerobic glycolysis and the Warburg effect [35]. In accordance, excessive acidic metabolites were extruded into the extracellular, acidifying the extracellular milieu with multiple membrane transport mechanisms to maintain relative week acidic extracellular pH (pHe) and basic intracellular pH (pHi) [36]. Under acidic condition, HSCs were activated with the phosphorylation of cellular signal-regulated kinase (ERK) and excreted osteopontin to promote the migration of HCC cells [6]. Because of their important role in progression of liver fibrosis and HCC, activated HSCs could be suitable target cells for liver fibrosis and HCC therapy. It is worth attempting to alter the weak acidic microenvironment to inhibit activated HSCs proliferation or promote apoptosis to treat liver fibrosis and liver cancer.

OA is a widespread natural plant and Chinese medicinal plant, used for prevention and treatment of clinical liver disease. Now, there are large number of oleanolic acid derivatives that have been synthesized and hopefully developed into clinical liver protection drug [20]. In our previous report, a variety of OA’s derivatives were designed, synthesized, and evaluated according to bio-activity, and a series of hepatoprotective lead compounds with high efficiency and low toxicity were screened out [16,18,19,25]. In this report, a series of OA-amino acid derivatives were designed and synthesized to improve biological activity and hydrophilicity. After introducing different amino acids in the 3-hydroxyl of OA using the ester condensation reaction, the ClogP of the derivatives reduced to a certain degree, which of glycine, alanine, and lysine conjugates was most significant. It was also in line with the theory of drug design and our previous reports, as well as others [22,23,24,25]. More importantly, most of the derivatives had some alkalinity with different cell inhibitory activities. The results suggested that the alkalinities of the derivatives were positively correlated with their activities, and OA-lysine conjugates (**3g**) could significantly promote apoptosis through the mitochondrial apoptotic pathway, and effectively reduce the enzymatic activity of ALT and AST in serum to exert hepatoprotective activity on CCl_4_ acute liver injury mice model. Thence, **3g** might induce apoptosis of HSCs by changing the extracellular acidic environment and regulating the protons transmembrane transport mechanism, which needs further research to be confirmed. It was noteworthy that OA’s derivatives, such as CPU-II2, CDDO-EA, and Oxy-Di-OA, could attenuate the expression of TNF-α, α-SMA and TGF-β, and improve histologic and serologic markers of fibrosis in CCl4-induced hepatic fibrosis in rats [19,37,38]. However, it needs to be verificated further as to whether **3g** exerted antifibrotic activity by the expression of related proteins. 

## 4. Materials and Methods

Reactions were monitored by TLC using silica gel-coated aluminum sheets (Qingdao Haiyang Chemical Co., Qingdao, China) and visualized in UV light (254 nm). ^1^H-NMR and ^13^C-NMR assays were recorded on a Bruker AVANCE 500 NMR spectrometer (Fällanden, Switzerland), and chemical shifts are reported in form of δ (ppm). Deuterated chloroform, deuterated methanol, or deuterated DMSO served as the solvent (Beijing InnoChem Science and Technology Co., Ltd., Beijing, China). HR-MS spectra were performed on high-resolution ESI mass spectrum (Solarix 9.4T, Bruker, Germany). Melting points were measured at a rate of 5 °C/min using an X-5 micro melting point apparatus (Beijing Tech Instrument Co., Ltd., Beijing, China). The specific rotation of the synthesized compounds was measured using P-1020 polarimeter (Jasco, Tokyo, Japan). Cellular morphologies were observed using an inverted fluorescence microscope (Olympus IX71, Tokyo, Japan). Mechanisms of apoptosis were detected by flow cytometry (BD FACSCanto II, San Jose, CA, USA). Silica-gel column chromatography was performed using 200–300 mesh silica gel. The yields were calculated based on the last step reaction. All solvents and chemicals used were analytical or high-performance liquid chromatography grade. The abilities of ALT and AST in serum were measured with assay kit (Nanjing Jiancheng Biotechnology Co., Ltd., Nanjing, China).

### 4.1. General Synthesis of Compounds ***3a***–***3j***

To a solution of the corresponding Cbz-(l)-amino acids (0.38 mmol), EDCI (0.50 mmol), and DMAP (0.025 mmol) in 20 mL dry DCM, compound 1 (0.25 mmol) was added. Additionally, the reaction mixture was stirred at room temperature overnight. Reaction was monitored by TLC. After completion of the reaction, the crude product was extracted with DCM. After drying the organic layer over anhydrous Na_2_SO_4_ and evaporating the solvent under vacuum, the crude product was purified by flash chromatography with dichloromethane–methanol (20:1) as eluent. The crude product and suiTable 10% Pd(OH)_2_/C were stirred overnight in methanol in a hydrogen atmosphere. The reaction solution was filtered and evaporated with vacuum. After separation by flash chromatography with dichloromethane–methanol (20:1) as eluent, the product was lyophilized.

**3a**: White solid, 44.2% yield, m.p. 203.1–204.0 °C, ${\left[\alpha \right]}_{D}^{25}$ = +24.9 (*c* = 1.0 mg/mL, MeOH), ^1^H-NMR (500 MHz, CDCl_3_) δ (ppm): 5.22 (brs, 1H, H-12), 4.56 (m, 1H, H-3), 3.45 (s, 2H, NH_2_CH_2_-), 2.82 (m, 1H, H-18), 0.92, 0.90 (brs, 12H, 4× -CH_3_), 2.50–1.00 (22H, methylene and methine of OA), 1.13, 0.86, 0.76 (s, each, 3H, 5× -CH_3_);^13^C-NMR (125 MHz, CDCl_3_) δ (ppm): 183.3 (-CO-), 173.6 (-CO-), 143.9 (-CH=C-), 122.5 (-CH=C-), 82.1 (-OCOCH-), 55.4, 47.7, 46.6, 44.5, 43.6, 41.8, 41.5, 39.4, 38.6, 37.9, 37.1, 33.9, 33.2, 32.8, 32.2, 30.8, 29.8, 28.2, 27.8, 26.1, 23.7, 23.5, 23.1, 18.3, 17.2, 16.8, 15.5. HR-MS (ESI) *m*/*z*: 514.38684 [M + H]^+^, calcd for C_32_H_52_NO_4_: 514.38181.

**3b**: White solid, 48.4% yield, m.p. 193.8–195.5 °C, ${\left[\alpha \right]}_{D}^{25}$ = +56.7 (*c* = 1.0 mg/mL, MeOH), ^1^H-NMR (500 MHz, CDCl_3_) δ (ppm): 5.27 (brs, 1H, H-12), 4.53 (m, 1H, H-3), 3.48 (m, 1H, NH_2_CH-), 2.82 (m, 1H, H-18), 1.61 (m, 3H, -CHCH_3_), 2.50–1.00 (22H, methylene and methine of OA), 1.13, 0.93, 0.92, 0.90, 0.75 (s, each, 3H, 5× -CH_3_), 0.86 (brs, 6H, 2× -CH_3_); ^13^C-NMR (125 MHz, CDCl_3_) δ (ppm): 183.2 (-CO-), 176.2 (-CO-), 144.0 (-CH=C-), 122.4 (-CH=C-), 81.8 (-OCOCH-), 55.4, 47.7, 46.6, 46.1, 41.8, 41.2, 39.4, 38.2, 38.0, 37.9, 37.1, 33.9, 33.2, 32.7, 32.6, 31.1, 30.8, 28.2, 27.8, 26.0, 23.7, 23.6, 23.5, 23.1, 18.3, 17.3, 16.9, 15.5. HR-MS (ESI) *m*/*z*: 528.40186 [M + H]^+^, calcd for C_33_H_54_NO_4_:528.39746.

**3c**: White solid, 41.9% yield, m.p. 190.5–191.7 °C, ${\left[\alpha \right]}_{D}^{25}$ = +37.5 (*c* = 1.0 mg/mL, MeOH), ^1^H-NMR (500 MHz, CDCl_3_) δ (ppm): 5.26 (brs, 1H, H-12), 4.54 (m, 1H, H-3), 4.00 (m, 1H, -NHCH-), 3.09 (m, 2H, -NHCH_2_-), 2.82 (m, 1H, H-18), 2.50–1.00 (26H, methylene and methine of OA, cyclomethylen of Proline), 1.12, 0.89, 0.76 (s, each, 3H, 3× -CH_3_), 0.92, 0.85 (brs, 12H, 4× -CH_3_); ^13^C-NMR (125 MHz, CDCl_3_) δ (ppm): 182.8 (-CO-), 173.7 (-CO-), 144.1 (-CH=C-), 122.1 (-CH=C-), 82.4 (-OCOCH-), 59.0, 55.3, 47.6, 46.5, 46.0, 41.7, 41.1, 39.3, 38.0, 37.9, 37.8, 37.0, 33.9, 33.1, 32.6, 32.2, 30.8, 30.3, 28.2, 27.8, 25.9, 25.4, 25.1, 23.7, 23.4, 23.0, 18.2, 17.1, 16.7, 15.4. HRMS (ESI) *m*/*z*: 554.41656 [M + H]^+^, calcd for C_35_H_56_NO_4_: 554.41311.

**3d**: White solid, 43.3% yield, m.p. 205.1–206.5 °C, ${\left[\alpha \right]}_{D}^{25}$ = +50. 6 (*c* = 1.0 mg/mL, MeOH), ^1^H-NMR (500 MHz, CDCl_3_) δ (ppm): 5.27 (brs, 1H, H-12), 4.51 (m, 1H, H-3), 3.33 (m, 1H, NH_2_CH-), 2.82 (m, 1H, H-18), 2.50–0.80 (24H, methylene and methine of OA and Leucine), 1.13, 0.94, 0.88, 0.86, 0.75 (s, each, 3H, 5× -CH_3_), 0.93, 0.90 (brs, 12H, 4× -CH_3_); ^13^C-NMR (125 MHz, CDCl_3_) δ (ppm): 184.0 (-CO-), 172.3 (-CO-), 143.8 (-CH=C-), 122.6 (-CH=C-), 81.7 (-OCOCH-), 55.5, 47.7, 46.7, 46.0, 41.7, 41.1, 39.4, 38.2, 37.9, 37.7, 37.1, 33.9, 33.2, 32.7, 32.6, 30.8, 28.3, 27.8, 26.1, 25.2, 25.1, 23.7, 23.5, 23.4, 23.1, 22.9, 22.6, 18.3, 17.3, 16.9, 15.5. HR-MS (ESI) *m*/*z*: 570.44916 [M + H]^+^, calcd for C_36_H_60_NO_4_: 570.44441.

**3e**: White solid, 45.2% yield, m.p. 206.9–207.7 °C, ${\left[\alpha \right]}_{D}^{25}$ = +49.4 (*c* = 1.0 mg/mL, MeOH), ^1^H-NMR (500 MHz, CDCl_3_) δ (ppm): 5.27 (brs, 1H, H-12), 4.54 (m, 1H, H-3), 2.82 (m, 1H, H-18), 2.50–1.00 (29H, methylene and methine of OA and Isoleucine), 1.13, 0.94, 0.92, 0.88, 0.86, 0.75 (s, each, 3H, 6× -CH_3_), 0.90 (brs, 6H, 2× -CH_3_); ^13^C-NMR (125 MHz, CDCl_3_) δ (ppm): 183.6 (-CO-), 173.3 (-CO-), 143.8 (-CH=C-), 122.6 (-CH=C-), 82.0 (-OCOCH-), 67.0, 55.5, 47.7, 46.7, 46.0, 41.8, 41.1, 39.4, 38.2, 38.1, 37.8, 37.7, 37.1, 34.0, 33.2, 32.7, 32.6, 30.8, 30.2, 28.3, 27.8, 26.0, 24.5, 23.7, 23.5, 23.1, 18.3, 17.3, 17.0, 15.5, 11.7; HR-MS (ESI) *m*/*z*: 570.44904 [M + H]^+^, calcd for C_36_H_60_NO_4_: 570.44441.

**3f**: White solid, 52.8% yield, m.p. 174.4–174.9 °C, ${\left[\alpha \right]}_{D}^{25}$ = +74.8 (*c* = 1.0 mg/mL, MeOH), ^1^H-NMR (500 MHz, CDCl_3_) δ (ppm): 5.27 (brs, 1H, H-12), 4.55 (m, 1H, H-3), 2.82 (m, 1H, H-18), 2.50–0.80 (30H, methylene and methine of OA, methyl and methylene of Valine), 1.13, 0.94, 0.92, 0.88, 0.86, 0.75 (s, each, 3H, 5× -CH_3_), 0.89 (brs, 6H, 2× -CH_3_); ^13^C-NMR (125 MHz, CDCl_3_) δ (ppm): 183.5 (-CO-), 173.7 (-CO-), 143.9 (-CH=C-), 122.5 (-CH=C-), 81.9 (-OCOCH-), 69.4, 55.5, 47.7, 46.6, 46.0, 41.8, 41.1, 39.4, 38.2, 37.9, 37.8, 37.1, 34.0, 33.2, 32.7, 32.6, 30.8, 28.4, 28.3, 27.8, 26.0, 24.0, 23.7, 23.5, 23.1, 18.3, 17.3, 17.0, 16.9, 15.5; HR-MS (ESI) *m*/*z*: 556.43329 [M + H]^+^, calcd for C_35_H_58_NO_4_: 556.42876.

**3g**: White solid, 40.9% yield, m.p. 168.4–169.5 °C, ${\left[\alpha \right]}_{D}^{25}$ = +84.9 (*c* = 1.0 mg/mL, MeOH), ^1^H-NMR (500 MHz, MeOD-d4:DMSO-d6 = 1:1) δ (ppm): 5.28 (brs, 1H, H-12), 4.57 (m, 1H, H-3), 3.49 (m, 1H, -NHCH-), 2.82 (m, 1H, H-18), 2.50–1.00 (35H, methylene and methine of OA and Lysine), 1.14, 0.94, 0.93, 0.90, 0.85, 0.83, 0.76 (s, each, 3H, 7× -CH_3_); ^13^C-NMR (125 MHz, MeOD-d4:DMSO-d6 = 1:1) δ (ppm):178.2 (-CO-), 173.5 (-CO-), 144.9 (-CH=C-), 124.6 (-CH=C-), 82.7 (-OCOCH-), 67.3, 66.9, 56.3, 55.7, 47.8, 47.0, 42.8, 42.7, 39.1, 38.8, 38.0, 34.7, 33.8, 33.7, 33.6, 31.7, 28.9, 28.6, 27.9, 26.6, 24.6, 24.4, 24.3, 24.1, 23.9, 20.7, 19.2, 17.9, 17.6, 16.1, 14.4. HR-MS (ESI) *m*/*z*: 585.45966 [M + H]^+^, calcd for C_36_H_60_N_2_O_4_: 585.45531.

**3h**: White solid, 46.1% yield, m.p. 152.5–153.0 °C, ${\left[\alpha \right]}_{D}^{25}$ = +78.5 (*c* = 1.0 mg/mL, MeOH), ^1^H-NMR (500 MHz, CDCl_3_) δ (ppm): 7.30, 7.29, 7.27, 7.23, 7.22 (s, each, 1H, -C_6_H_5_), 5.27 (brs, 1H, H-12), 4.53 (m, 1H, H-3), 3.77 (m, 1H, -CHNH_2_), 2.84 (m, 1H, H-18), 2.50–1.00 (24H, methylene and methine of OA and Phenylalanine), 1.13, 0.90, 0.83, 0.80, 0.75 (s, each, 3H, 5× -CH_3_), 0.92 (brs, 6H, 2× -CH_3_); ^13^C-NMR (125 MHz, CDCl_3_) δ (ppm): 183.5 (-CO-), 174.4 (-CO-), 143.9 (-CH=C-), 137.2, 129.6, 129.5, 128.8, 128.7, 127.0 (-C_6_H_5_), 122.5 (-CH=C-), 82.1 (-OCOCH-), 55.9, 55.4, 47.7, 46.6, 46.0, 41.7, 41.0, 39.4, 38.2, 37.9, 37.1, 34.0, 33.2, 32.7, 32.6, 30.8, 28.2, 27.8, 27.6, 26.0, 23.7, 23.5, 23.1, 18.3, 17.3, 16.9, 15.5; HRMS (ESI) *m*/*z*: 604.43359 [M + H]^+^, calcd for C_39_H_58_NO_4_: 604.42876.

**3i**: White solid, 54.7% yield, m.p. 174.7–175.8 °C, ${\left[\alpha \right]}_{D}^{25}$ = +181.2 (*c* = 1.0 mg/mL, MeOH), ^1^H-NMR (500 MHz, CDCl_3_) δ (ppm): 5.27 (brs, 1H, H-12), 4.57 (m, 1H, H-3), 3.38 (m, 2H, -NHCH_2_-), 2.82 (m, 1H, H-18), 2.45 (m, 3H, -NHCH_3_), 2.50–1.00 (23H, methylene and methine of OA), 1.13, 0.93, 0.92, 0.90, 0.86, 0.84, 0.76 (s, each, 3H, 7× -CH_3_); ^13^C-NMR (125 MHz, CDCl_3_) δ (ppm): 182.8 (-CO-), 171.8 (-CO-), 144.1 (-CH=C-), 122.3 (-CH=C-), 81.9 (-OCOCH-), 55.4, 52.1, 47.7, 46.6, 46.1, 41.8, 41.2, 39.4, 38.2, 37.9, 37.1, 35.6, 34.0, 33.3, 32.7, 32.6, 30.8, 28.2, 27.9, 26.0, 23.8, 23.5, 23.2, 18.3, 17.3, 16.8, 15.5; HR-MS (ESI) *m*/*z*: 528.47595 [M + H]^+^, calcd for C_33_H_54_NO_4_: 528.39746.

**3j**: White solid, 50.1% yield, m.p. 198.5–199.3 °C, ${\left[\alpha \right]}_{D}^{25}$ = +74.4 (*c* = 1.0 mg/mL, MeOH), ^1^H-NMR (500 MHz, CDCl_3_) δ (ppm): 6.79 (m, 1H, -NH-), 5.26 (brs, 1H, H-12), 4.56 (m, 1H, H-3), 4.26 (m, 1H, -NHCH-), 2.82 (m, 1H, H-18), 2.50–1.00 (23H, methylene and methine of OA and Pyroglutamate), 1.12, 0.93, 0.92, 0.90, 0.87, 0.86, 0.75 (s, each, 3H, 7× -CH_3_); ^13^C-NMR (125 MHz, CDCl_3_) δ (ppm): 183.5 (-CO-), 183.4 (-CO-), 171.7 (-CO-), 143.9 (-CH=C-), 122.4 (-CH=C-), 82.6 (-OCOCH-), 56.2, 55.4, 47.7, 46.6, 46.0, 41.7, 41.1, 39.4, 38.1, 37.9, 37.1, 33.9, 33.2, 32.7, 32.6, 30.8, 29.7, 28.3, 27.8, 26.0, 25.1, 23.7, 23.6, 23.5, 23.0, 18.3, 17.2, 16.9, 15.5; HR-MS (ESI) *m*/*z*: 568.39685 [M + H]^+^, calcd for C_35_H_54_NO_5_: 568.39237.

### 4.2. Cell Viability Assay

Cell viability was evaluated using an MTT assay. HSCs, HepG2, Bel-7402, and L02 cells were seeded, respectively, in 96-well plates at a density of 3 × 10^3^ cells per-well and stabilized at 37 °C for 24 h in a humidified atmosphere with 5% CO_2_. The growing cells were exposed to various concentrations of the tested drugs and incubated for 72 h (37 °C, 5% CO_2_). The MTT solution (20 μL, 5 mg/mL) was added to each well and incubated for 4 h. After removing the media, formazan crystals were dissolved with DMSO (150 μL). After shaking for 10 min, the absorbance was quantified at 490 nm. Wells containing no drugs were used as blanks. The IC_50_ values were defined as the concentration of compounds that produced a 50% reduction of surviving cells and were calculated using the Logit-method. The inhibitory rate was calculated in the following Equation (1):

Inhibition rate (%) = (1 − OD _Sample group_/OD _Control group_) × 100% (1)

### 4.3. Giemsa Staining Assay

HSCs cells in logarithmic growth phase were cultured in 6-well plates at a density of 3 × 10^4^ cells/mL for 24 h at 37 °C in a humidified atmosphere with 5% CO_2_. Then, each group was treated with **3g** at various concentrations (0, 1.5, 3.0, and 6.0 µM) for 72 h. The cell culture medium was discarded. Additionally, the cells were washed twice with PBS, kept in PBS/ethanol (1:1) for 2 min, and fixated in ethanol for 10 min. After removing the ethanol, the cells were stained with giemsa staining for 2 min and observed under inverted phase-contrast microscope at a magnification of 400×.

### 4.4. DAPI Staining

HSCs cells in logarithmic growth phase were cultured in 6-well plates at a density of 3 × 10^4^ cells/mL for 24 h at 37 °C in a humidified atmosphere with 5% CO_2_. Additionally, each group was treated with different concentrations of **3g** (0, 1.5, 3.0, and 6.0 µM) for 72 h. Cell culture medium was discarded, and the cells were washed twice with PBS. The cells were fixed with 4% paraformaldehyde (pH 7.4) for 15 min and then washed twice with PBS. With an excitation wavelength of 470 nm, 4′,6-diamidino-2-phenylindole (DAPI) staining was then performed for 2 min, and nuclear fragments were observed using inverted phase-contrast microscope at a magnification of 400×.

### 4.5. Annexin V-FITC/PI Staining Assay

HSCs cells in logarithmic growth phase were cultured in 6-well plates at a density of 3 × 10^4^ cells/mL for 24 h at 37 °C in a humidified atmosphere with 5% CO_2_. HSCs cells were treated with **3g** at various concentrations (0, 1.5, 3.0, and 6.0 µM) for 72 h. Then, cells were collected, washed with annexin-binding buffer, and stained with annexin V-FITC and PI for 15 min in the dark. After that, the samples were analyzed by flow cytometry (BD FACSCalibur, San Jose, CA, USA).

### 4.6. Mitochondrial Membrane Potential Assay

HSCs cells in logarithmic growth phase were seeded in 6-well culture plate at a density of 3 × 10^4^ cells/mL for 24 h at 37 °C in incubator with 5% CO_2_. After treatment with different concentrations of **3g** (0, 1.5, 3.0, and 6.0 μM) for 72 h, cells were collected and washed twice in cold PBS, then resuspended in 1 mL cell culture medium with Rh123 (10 μg/mL) and incubated for 30 min at 37 °C. After being washed twice with cold PBS, cells were immediately analyzed by the flow cytometer at 488 nm excitation wavelength.

### 4.7. Intracellular Free Ca^2+^ Levels Assay

HSCs cells in logarithmic growth phase were cultured in 6-well plates at a density of 3 × 10^4^ cells/mL for 24 h at 37 °C in incubator with 5% CO_2_. After treatment with different concentrations of **3g** (0, 1.5, 3.0, and 6.0 μM) for 72 h, cells were collected and washed twice with cold PBS, then resuspended in HBSS buffer with 10 μM Fluo-3AM and incubated for 30 min at 37 °C in the dark. Intracellular Ca^2+^ levels were analyzed by the flow cytometer at 488 nm excitation wavelength.

### 4.8. In Vivo Activity on CCl_4_ Acute Liver Injury Mice Model

The twelve mice were randomly divided into the control group, model group, administration of OA group, and administration of **3g** group. In the administration of OA and **3g** group, the mice were fed with OA and **3g** suspension with 0.5% CMC-Na at a dose of 20 mg/kg body weight. The control group and model group were intragastrically administered with equal volume of 0.5% CMC-Na; during administration for 8 days, the general activity, fur, and feces of mice were observed daily. After administration for 1 h on the 8th day, the other groups were intraperitoneally injected with 10mL/kg 0.2% of the carbon tetrachloride (CCl_4_) olive oil solution, while the control group was intraperitoneally injected with olive oil solution. After 16 h of injury, the serum was prepared by centrifugation at 3000 rpm by orbital blood. The ability of ALT and AST was measured according to the instructions of the assay kit. The liver tissues of each group were taken out and the morphological differences were observed. The large lobe of liver was taken for pathological examination with HE staining under an optical microscope (×100).

### 4.9. Statistical Analysis

All results were expressed as means ± standard derivation (SD) of three independent experiments. Statistical significance was evaluated with the one-way ANOVA using SPSS 17.0 (SPSS Inc., Kraków, Poland). *p*-values < 0.05 were considered significant, *p*-values < 0.01 were considered very significant, and *p*-values < 0.001 were considered extremely significant.

## 5. Conclusions

In this paper, a series of novel OA-amino acids analogues was designed and synthesized to introduce different amino acids in the 3-hydroxyl of OA to enhance hydrophilicity, alkalinity, and biological activity. Because of their clinical roles in liver fibrosis and liver cancer, activated HSCs were selected as target cells to evaluate their activity in vitro. We found that OA-lysine derivative (**3g**) could improve hydrophilic and alkaline of OA, induce HSCs apoptosis via inducing MMP depolarization, and increase intracellular Ca^2+^ levels. Additionally, **3g** also displayed a better hepatoprotective effect than OA against the acute liver injury induced by CCl_4_ in mice. The results suggested basic amino acid (lysine) could effectively enhance OA’s hydrophilicity, alkalinity, and hepatoprotective activity in vitro and in vivo, which might be likely associated with increasing bioavailability and the alteration of the extracellular, weak acidic microenvironment with further verification. Therefore, OA-lysine derivative (**3g**) has the potential to be developed as an agent with hepatoprotective activity.

## Figures and Tables

**Table 1:** In vitro biological activities of OA-amide acids derivatives.

Compounds	IC_50_ (μM)			
	HSCs	HepG2	Bel-7402	L02
**OA**	>50 ^a^	>50	>50	>50
**3a**	13.58 ± 1.12	11.50 ± 0.95	12.09 ± 1.32	>25 ^b^
**3b**	9.92 ± 1.05	12.17 ± 1.48	>25	>50
**3c**	15.55 ± 2.31	11.05 ± 0.63	10.49 ± 1.49	10.08 ± 0.56
**3d**	>25	>25	>25	>25
**3e**	24.53 ± 1.99	18.00 ± 1.78	18.66 ± 1.63	>25
**3f**	19.61 ± 3.20	18.64 ± 2.65	>25	15.22 ± 1.20
**3g**	3.46 ± 1.16	3.67 ± 0.61	4.70 ± 0.75	12.31 ± 2.34
**3h**	14.76 ± 0.80	11.49 ± 0.94	8.50 ± 1.65	>50
**3i**	7.30 ± 1.58	>25	11.83 ± 0.83	>50
**3j**	>25	23.90 ± 2.65	>25	>50

^a^ >50 means IC_50_ value was more than 50 µM, ^b^ >25 means IC_50_ value was more than 25 µM, and it had shown no significant activity. The values (µM) represent the mean ± SD from three independent experiments.
